# Galectin-3 gene deletion results in defective adipose tissue maturation and impaired insulin sensitivity and glucose homeostasis

**DOI:** 10.1038/s41598-020-76952-z

**Published:** 2020-11-18

**Authors:** Claudia Blasetti Fantauzzi, Carla Iacobini, Stefano Menini, Martina Vitale, Gian Pio Sorice, Teresa Mezza, Saverio Cinti, Andrea Giaccari, Giuseppe Pugliese

**Affiliations:** 1 Department of Clinical and Molecular Medicine, “La Sapienza” University, Via di Grottarossa, 1035-1039, 00189 Rome, Italy; 2 Centre for Endocrine and Metabolic Diseases, Fondazione Policlinico Universitario A. Gemelli IRCCS, Catholic University, Rome, Italy; 3 Department of Experimental and Clinical Medicine, Center of Obesity, University of Ancona (Politecnica delle Marche), Ancona, Italy

**Keywords:** Diseases, Endocrinology, Pathogenesis

## Abstract

Adiposopathy is a pathological adipose tissue (AT) response to overfeeding characterized by reduced AT expandability due to impaired adipogenesis, which favors inflammation, insulin resistance (IR), and abnormal glucose regulation. However, it is unclear whether defective adipogenesis causes metabolic derangement also independently of an increased demand for fat storage. As galectin-3 has been implicated in both adipocyte differentiation and glucose homeostasis, we tested this hypothesis in galectin-3 knockout (*Lgal3*^−/−^) mice fed a standard chow. In vitro*, Lgal3*^−/−^ adipocyte precursors showed impaired terminal differentiation (maturation). Two-month-old *Lgal3*^−/−^ mice showed impaired AT maturation, with reduced adipocyte size and expression of adipogenic genes, but unchanged fat mass and no sign of adipocyte degeneration/death or ectopic fat accumulation. AT immaturity was associated with AT and whole-body inflammation and IR, glucose intolerance, and hyperglycemia. Five-month-old *Lgal3*^−/−^ mice exhibited a more mature AT phenotype, with no difference in insulin sensitivity and expression of inflammatory cytokines versus WT animals, though abnormal glucose homeostasis persisted and was associated with reduced β-cell function. These data show that adipogenesis capacity per se affects AT function, insulin sensitivity, and glucose homeostasis independently of increased fat intake, accumulation and redistribution, thus uncovering a direct link between defective adipogenesis, IR and susceptibility to diabetes.

## Introduction

The ongoing epidemics of obesity and diabetes have focused attention on adipose tissue (AT)^1^, as fat accumulation is causally related to insulin resistance (IR) and type 2 diabetes^2^. However, the existence of both metabolically healthy obese individuals and unhealthy insulin-resistant lean (metabolically obese) subjects^3^ indicates that it is not just a matter of absolute quantity of fat and shifts the focus on dysfunction of AT and remodeling of AT depots, i.e., the so-called adiposopathy^4,5^. Adiposopathy is a pathological AT response to positive caloric balance in susceptible individuals contributing to increased cardiometabolic risk. It is characterized by limited AT plasticity, i.e., reduced capacity to expand and accommodate the surplus of energy^6^. Expansion of AT relies upon adipogenesis, the process by which pluripotent mesenchymal stem cells commit to the adipose lineage to become pre-adipocytes, which then differentiate into adipocytes by undergoing mitotic clonal expansion, followed by growth arrest and terminal differentiation with acquisition of a mature phenotype, i.e., expression of adipocyte genes and accumulation of triglycerides^7^. The overall AT capacity to store excess fat is dependent upon the ability of increasing both adipocyte number and size without achieving a critical cell volume^4^. When this capacity is overwhelmed, adipocytes degenerate and die by pyroptosis^8^, with consequent fibrosis and macrophage infiltration^9^. These processes ultimately lead to ectopic fat accumulation, tissue and systemic inflammation and IR, and development of type 2 diabetes^10,11^. While the role of adiposopathy in the pathological response to positive caloric balance is well established, it is unclear whether impaired adipogenesis per se may affect insulin sensitivity and glucose homeostasis, independently of an increased demand for fat storage.

Among the factors that control adipogenesis by regulating the various steps in the commitment and differentiation programs^7^, there is galectin-3^12^, a multifunctional protein involved in many physiological and pathological processes^13,14^. In human AT, galectin-3 was found to be expressed also by adipocytes^15^, where it is modulated during cell differentiation^16^. Moreover, Baek et al. reported that galectin-3 induces nuclear translocation and activation of peroxisome proliferator-activated receptor (PPAR) γ in 3T3-L1 cells, whereas both mouse embryonic fibroblasts isolated from galectin 3 knockout (*Lgals3*^−/−^) mice and stably galectin-3-silenced 3T3-L1 cells show delayed adipogenic differentiation^17^. They also found that aged (17-month-old) *Lgals3*^−/−^ mice display reduced body weight associated with decreased epididymal AT size and expression of adipogenic and lipogenic genes compared with wild-type (WT) mice^17^. Finally, other studies reported unchanged weight gain associated with either increased blood glucose levels in younger (3-to-5-month-old)^18,19^ or improved glucose regulation in older (8-month-old)^20^ *Lgals3*^−/−^ versus WT mice.

To date, the relationship between decreased AT expression of adipogenic and lipogenic genes^17^ and impaired glucose homeostasis associated with unchanged body weight^18,19^ in *Lgals3*^−/−^ mice fed a standard chow remains unexplored. The aim of this study was to gain pathophysiological insights into the role of AT dysfunction due to defective adipogenesis in the impaired glucose regulation occurring in *Lgals3*^−/−^ mice in the absence of an increased demand for fat storage. To this end, the *Lgals3*^−/−^ mouse appears to be a suitable model for testing the working hypothesis that impaired adipogenesis may directly affect glucose homeostasis and contribute to metabolic derangement independently of environmental obesogenic or diabetogenic stimuli.

## Results

### In vitro* studies*

#### Adipogenic differentiation of adipocyte precursors

**Figure 1:**
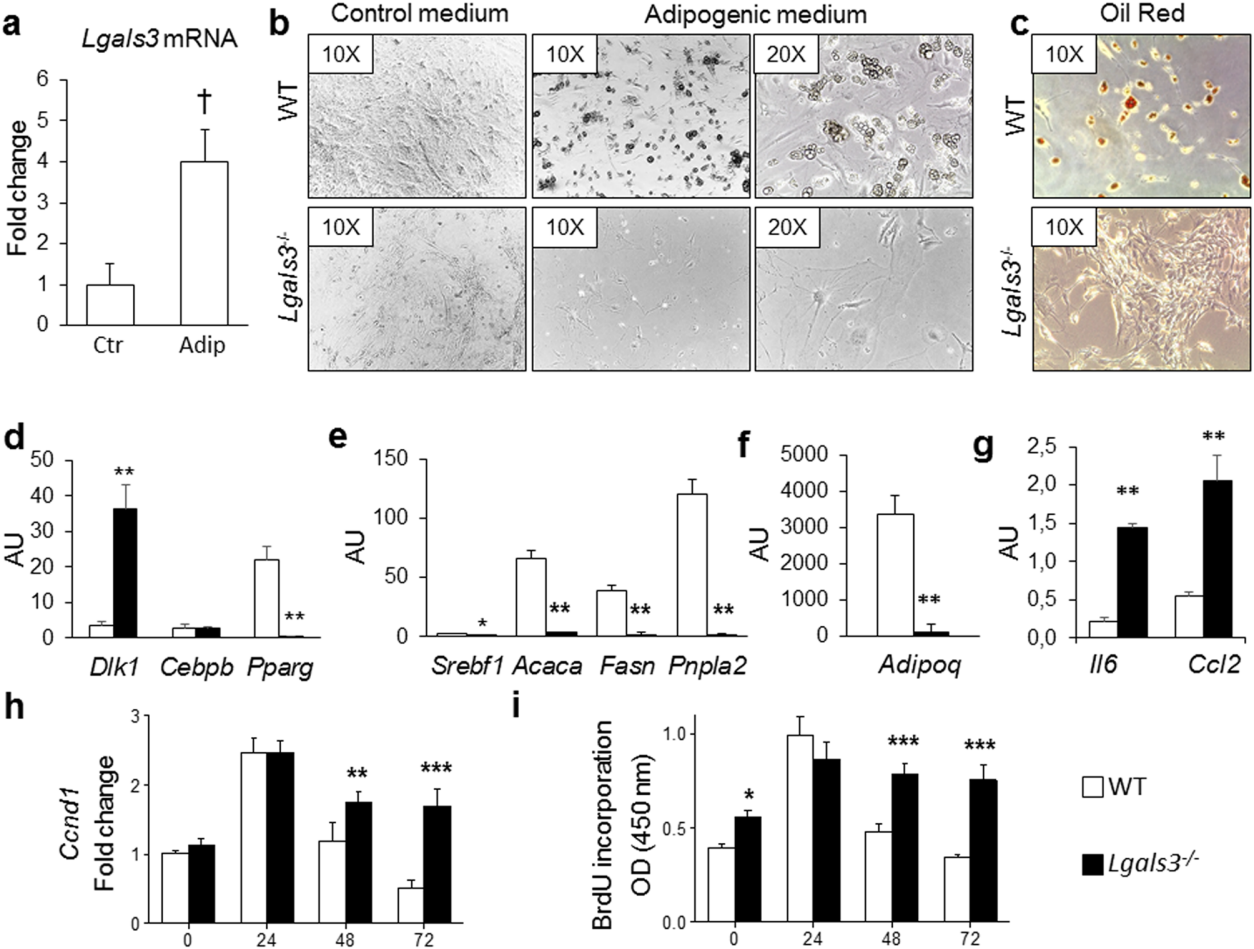
In vitro adipogenic differentiation of SVF cells. *Lgals3* mRNA levels from SVF cells of WT mice cultured in normal control (Ctr) or adipogenic (Adip) medium (**a**); representative images of adipogenic differentiation of SVF cells isolated from SAT of WT and *Lgals3*^−/−^ mice, cultured in control or adipogenic medium for 14 days (original magnification, 10X or 20X) (**b**); oil-Red-O staining of WT and *Lgals3*^−/−^ SVF cells induced to differentiation (**c**); qRT-PCR analysis of genes involved in adipogenesis (*Dlk1*, *Cebpb*, and *Pparg*) (**d**), lipid metabolism (*Srebf1*, *Acaca*, *Fasn*, and *Pnpl* + *a2*) (**e**), adipokine synthesis (*Adipoq*) (**f**), inflammation (*Il6* and *Ccl2*) (**g**), and proliferation (*Ccnd1*) (**h**); and analysis of BrdU incorporation (**i**) from SVF cells induced to differentiation of WT and *Lgals3*^−/−^ mice. Values represent the mean ± SD of two independent experiments (n = 3 mice per group per experiment). †*P* < 0.01 versus control medium; *P < 0.05, **P < 0.01, or ***P < 0.001 versus WT.

Adipose-derived stromal vascular fraction (SVF) precursor cells were isolated from subcutaneous AT (SAT) of WT and *Lgals3*^−/−^ and exposed to an adipogenic medium. Under these conditions, *Lgals3* expression increased in SVF cells from WT mice (Fig. 1a). The SVF cells from *Lgals3*^−/−^ mice, though replicated well in culture, showed impaired adipogenesis, maintaining an immature (fibroblast-like) phenotype (Fig. 1b) with negative Oil-Red-O staining even after 14 days (Fig. 1c). Conversely, the SVF cells from WT mice differentiated into round shaped adipocytes (Fig. 1b), filled with Oil-Red-O positive lipid droplets (Fig. 1c). Consistently, *Lgals3*^−/−^ cells showed higher Preadipocyte factor 1 or Delta Like Non-Canonical Notch Ligand (*Dlk1*) (Fig. 1d) and lower PPARγ (*Pparg*), but unchanged Ccaat-Enhancer-Binding Protein (CEBP) β (*Cebpb*) gene expression levels (Fig. 1d), as compared with WT cells. The mRNA levels of the genes involved in lipid metabolism Adipose Triglyceride Lipase or Patatin Like Phospholipase Domain Containing 2 (*Pnpla2*), Sterol Regulatory Element Binding Transcription Factor 1 (*Srebf1*), Acetyl-CoA Carboxylase α (*Acaca*), Fatty Acid Synthase (*Fasn*) (Fig. 1e) and the adiponectin gene (*Adipoq*) (Fig. 1f) were also lower, whereas those of the inflammatory markers interleukin (IL)-6 (*Il6)* and monocyte chemoattractant protein 1 or C–C Motif Chemokine Ligand 2 (*Ccl2*) were higher (Fig. 1g) in *Lgals3*^−/−^ versus WT cells. In addition, the mitotic clonal expansion following adipogenesis induction was abnormally regulated in *Lgals3*^−/−^ SVF cells. In fact, the expression of the gene coding for the cell cycle regulator cyclin D1 (*Ccnd1*), which is required for G1/S transition and is degraded as the cell enters the S phase (Fig. 1h), and the incorporation of the proliferation marker 5-bromo-2′-deoxyuridine (BrdU) (Fig. 1i) showed little or no decrease, respectively, 48 and 72 h after exposure to adipogenic medium in SVF cells from *Lgals3*^−/−^ mice, at variance with those from wild type animals. In addition, baseline BrUD incorporation was higher in *Lgals3*^−/−^ versus wild type SVF cells.

#### Lipid metabolism in mature adipocytes

To rule out the possibility that the reduced lipid accumulation detected in adipocytes derived from *Lgals3*^−/−^ SVF cells was due impaired lipid metabolism instead of defective adipogenesis, *Lgals3* was silenced in mature adipocytes. No differences were observed in the gene expression for lipogenic and lipolytic enzyme between silenced and non-silenced cells (Supplementary Fig. 2).

### In vivo*/*ex vivo* studies*

The growth curve did not differ between the two genotypes throughout the study (Supplementary Fig. 1a), as did food intake (not shown). Conversely, fasting plasma glucose levels showed a trend toward an increase in *Lgals3*^−/−^*Lgals3*^−/−^ versus WT mice, which became significant at 3 months of age (Supplementary Fig. 1b). The animals were studied at 2 and 5 months of age.

### Two-month-old mice

#### AT phenotype

**Figure 2:**
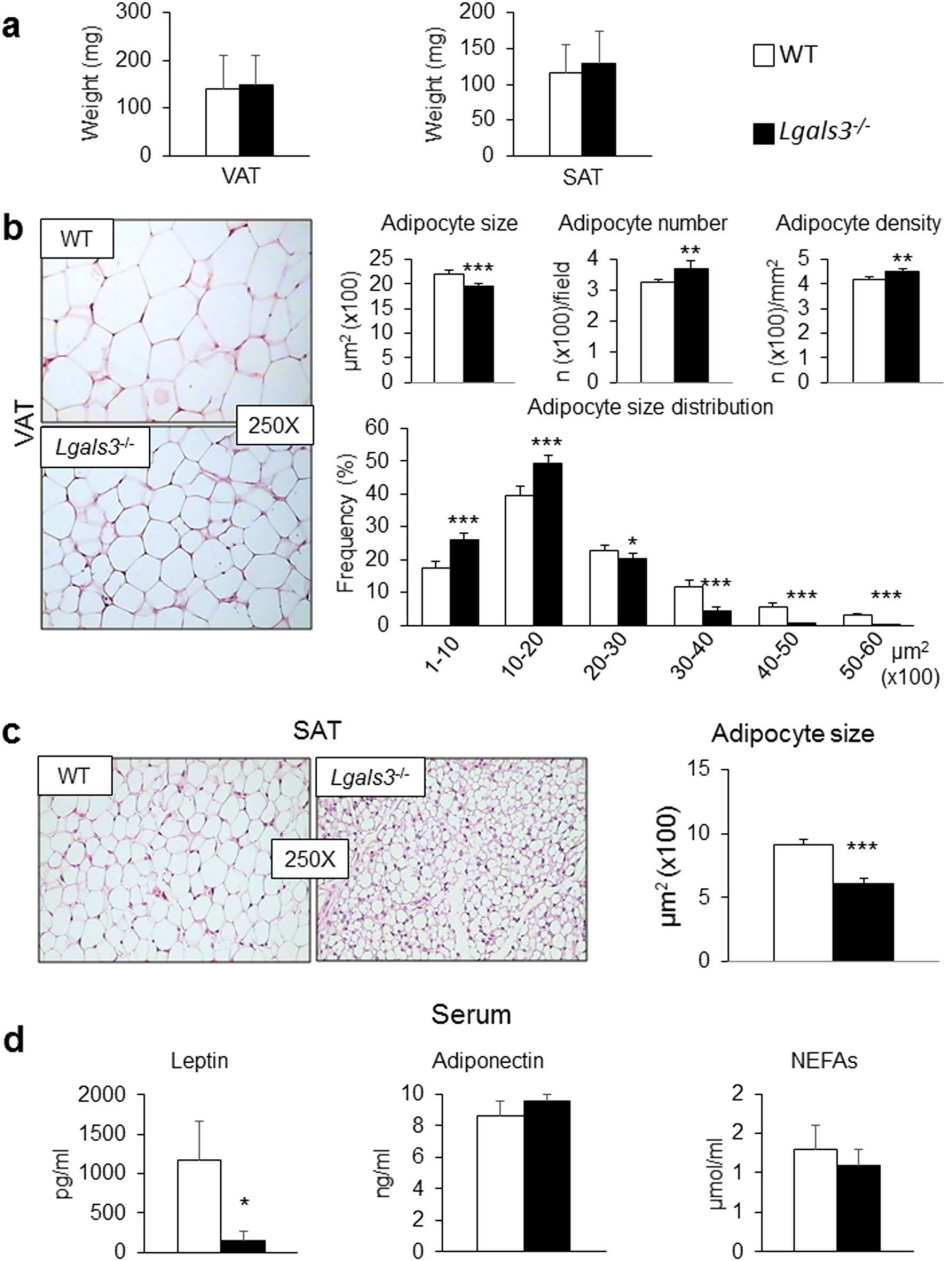
AT phenotype and serum levels of adipokines and NEFAs in WT and *Lgals3*^−/−^ mice aged 2 months. Weight of VAT and SAT (**a**); representative haematoxylin–eosin images of VAT (**b**) and SAT (**c**) (original magnification, 250X), quantification of size of adipocytes from VAT (**b**) and SAT (**c**), and number, density and size distribution of adipocytes from VAT (**b**) from WT and *Lgals3*^−/−^ mice; and serum levels of leptin, adiponectin and NEFAs in WT and *Lgals3*^−/−^ mice (**d**). Values represent the mean ± SD (n = 6 mice per genotype). White bars = WT mice; black bars = *Lgals3*^−/−^ mice; *P < 0.05, **P < 0.01 or ***P < 0.001 versus WT.

*Lgals3*^−/−^ mice showed no change in visceral AT (VAT) or SAT mass compared to coeval WT mice (Fig. 2a). No obvious sign of adipocyte degeneration was observed in VAT from *Lgals3*^−/−^ mice (Fig. 2b). Morphometric analysis revealed a significant decrease in average adipocyte size in VAT (Fig. 2b) and particularly in SAT (Fig. 2c) of *Lgals3*^−/−^ mice. Moreover, VAT from *Lgals3*^−/−^ mice showed a higher number and density of adipocytes as well as a change in adipocyte size distribution, with a higher frequency of smaller adipocytes (Fig. 2b). Furthermore, the transcriptional levels of genes involved in adipogenesis (*Pparg* and CEBP α [*Cebpa*] in SAT and VAT), and lipogenesis (*Srebf1* in SAT and *Acaca* in VAT) as well as of genes coding for adipokines (leptin [*Lep*] in SAT and VAT and Adipsin or Complement Factor D [*Cdf*] only in SAT) were reduced in *Lgals3*^−/−^ versus WT mice (Table 1). Transcripts for *Pparg*, *Srebf1*, *Acaca*, and *Fasn* were reduced in brown AT (BAT) (Table 1). The expression levels of the “browning” genes Cell Death-Inducing DFFA-Like Effector A (*Cidea*) and PPARα (*Ppara*) were also lower in SAT from *Lgals3*^−/−^ versus WT mice. Furthermore, the gene expression for Uncoupling Protein 1 (*Ucp1*) was significantly reduced in BAT of *Lgals3*^−/−^ versus WT mice (Table 1). Circulating leptin was markedly reduced in *Lgals3*^−/−^ versus WT mice, whereas adiponectin and non-esterified fatty acid (NEFA) levels were unchanged (Fig. 2d).

**Table 1:** Adipogenesis, lipid metabolism and fibrosis in adipose tissue of 2-month-old mice.

	SAT		VAT		BAT	
	WT	*Lgals3*^−/−^	WT	*Lgals3*^−/−^	WT	*Lgals3*^−/−^
**Adipogenesis**						
*Cebpb*	0.91 ± 0.41	0.31 ± 0.16†	1.80 ± 1.48	1.77 ± 0.99	1.45 ± 0.47	0.91 ± 0.31
*Pparg*	0.61 ± 0.25	0.42 ± 0.29*	1.31 ± 0.68	0.96 ± 0.39*	1.03 ± 0.24	0.57 ± 0.16*
*Cebpa*	0.61 ± 0.24	0.27 ± 0.16*	1.04 ± 0.56	0.56 ± 0.17*	0.90 ± 0.13	0.71 ± 0.08
**Lipolysis**						
*Pnpla2*	0.47 ± 0.31	0.21 ± 0.10	0.81 ± 0.62	0.64 ± 0.39	0.93 ± 0.16	0.72 ± 0.15
**Lipogenesis**						
*Srebf1*	0.90 ± 0.39	0.49 ± 0.06*	1.07 ± 0.45	1.14 ± 0.32	0.93 ± 0.06	0.59 ± 0.04‡
*Acaca*	0.68 ± 0.39	0.63 ± 1.19	0.89 ± 0.32	0.54 ± 0.14*	1.03 ± 0.24	0.17 ± 0.04†
*Fasn*	0.68 ± 0.76	0.81 ± 1.31	1.09 ± 0.50	0.70 ± 0.68	0.93 ± 0.06	0.13 ± 0.04‡
*Fabp4*	0.54 ± 0.34	0.24 ± 0.09	0.80 ± 0.60	0.56 ± 0.25	1.03 ± 0.33	0.66 ± 0.09
**Adipokines**						
*Lep*	0.42 ± 0.33	0.10 ± 0.08*	0.56 ± 0.29	0.17 ± 0.12*	0.80 ± 0.21	0.44 ± 0.26
*Adipoq*	0.73 ± 0.28	0.45 ± 0.19	1.22 ± 0.64	0.81 ± 0.23	1.04 ± 0.40	0.80 ± 0.20
*Cfd*	0.83 ± 0.46	0.33 ± 0.12*	1.40 ± 1.02	0.87 ± 0.33	1.10 ± 0.37	0.87 ± 0.12
**Browning**						
*Cidea*	0.34 ± 0.37	0.08 ± 0.02*	0.87 ± 0.86	0.65 ± 0.85	0.95 ± 0.18	0.72 ± 0.19
*Ppara*	0.50 ± 0.36	0.13 ± 0.06*	1.59 ± 0.99	1.12 ± 0.73	1.17 ± 0.36	0.74 ± 0.25
*Ucp1*	N/A	N/A	N/A	N/A	0.91 ± 0.17	0.42 ± 0.16*
**Insulin signaling**						
*Slc2a4*	0.45 ± 0.38	0.06 ± 0.08*	1.35 ± 0.35	0.73 ± 0.22‡	0.97 ± 0.04	0.31 ± 0.08‡
*Insr*	0.70 ± 0.30	0.47 ± 0.15	1.10 ± 0.34	1.42 ± 0.39	1.04 ± 0.17	0.87 ± 0.04
*Irs1*	0.84 ± 0.23	0.50 ± 0.24*	1.02 ± 0.60	0.70 ± 0.25	1.46 ± 0.94	1.12 ± 0.24
**Fibrosis**						
*Fn1*	1.67 ± 1.73	0.64 ± 0.22	2.32 ± 1.72	3.65 ± 1.06	1.14 ± 0.17	0.94 ± 0.06
*Cola1a1*	3.32 ± 4.22	2.76 ± 2.96	1.44 ± 0.85	3.01 ± 1.75	0.92 ± 0.10	1.36 ± 0.17*
*Cola4a1*	0.49 ± 0.29	0.25 ± 0.10	0.84 ± 0.56	1.02 ± 0.66	0.97 ± 0.13	1.26 ± 0.01*
*Cola6a1*	0.58 ± 0.24	0.36 ± 0.12	1.06 ± 0.68	1.51 ± 1.28	0.98 ± 0.03	1.47 ± 0.41

Expression of genes involved in adipogenesis, white and brown/beige lipid metabolism, insulin signaling, and fibrosis in SAT, VAT and BAT from 2-month-old WT and *Lgals3*^−/−^ mice.

Values represent the mean ± SD (n = 7 per genotype). *P* values calculated by t-test between WT and *Lgals3*^−/−^: *P < 0.05; †*P* < 0.01 and ‡*P* < 0.001 versus the corresponding WT mice.

#### AT and systemic inflammation

**Figure 3:**
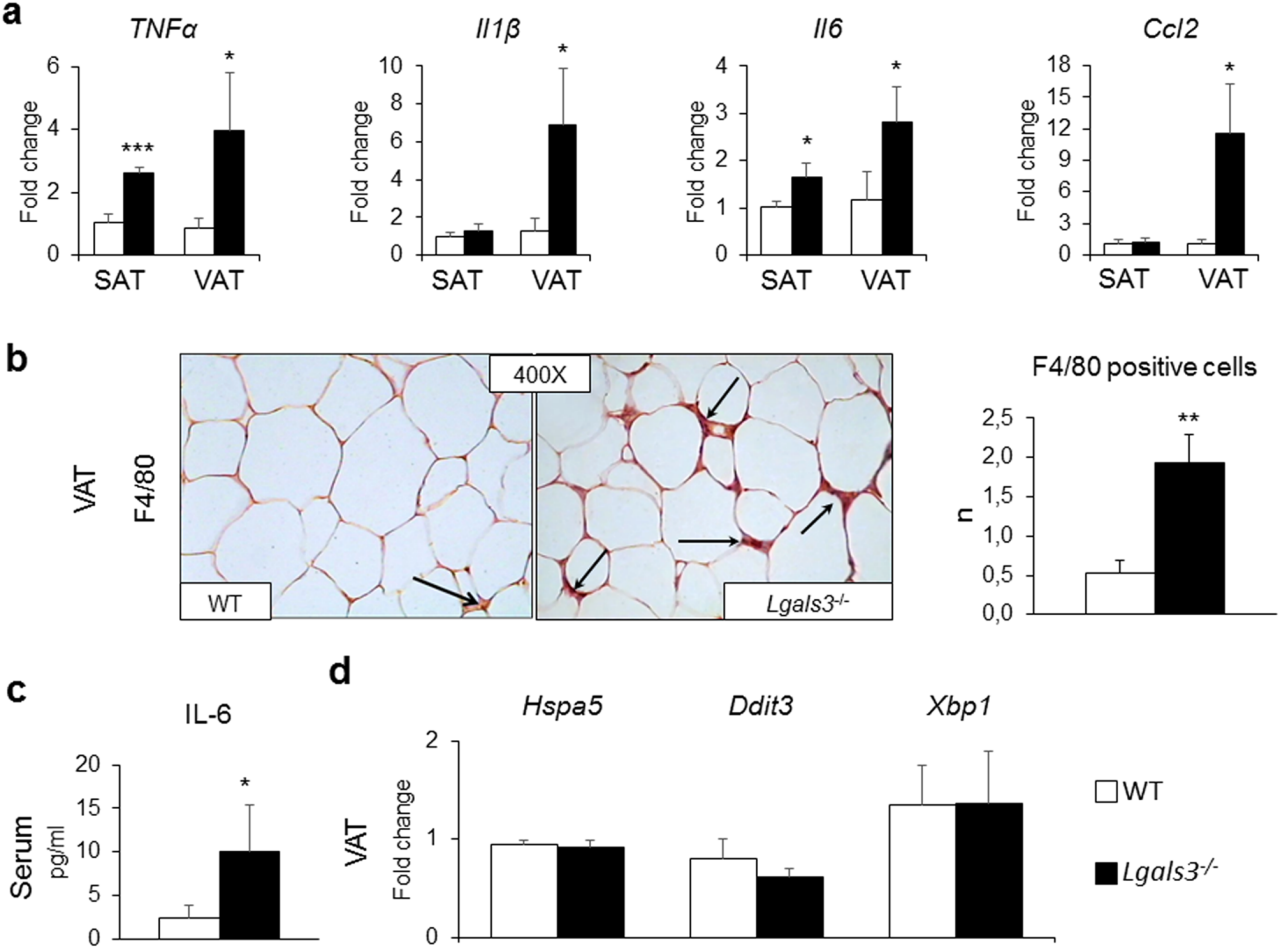
Tissue and systemic inflammation in WT and *Lgals3*^−/−^ mice aged 2 months. qRT-PCR analysis of the inflammatory markers *Tnfa*, *Il1b*, *Il6,* and *Ccl2* in SAT and VAT (**a**); representative immunohistochemical staining for F4/80 (original magnification, 400X; black arrows = macrophages), and measurement of positive cell number in VAT (**b**); serum levels of IL-6 (**c**); and qRT-PCR analysis of the ER stress markers *Hspa5*, *Ddit3*, and *Xbp1* in VAT (**d**) in WT and *Lgals3*^−/−^ mice. Values represent the mean ± SD (n = 6 mice per genotype). White bars = WT mice; black bars = *Lgals3*^−/−^ mice; *P < 0.05, **P < 0.01 or ***P < 0.001 versus WT.

The gene expression of tumor necrosis factor (TNF)-α *(Tnfa)* and *Il6* were significantly increased in both SAT and VAT, whereas the mRNA levels of IL-1β (*Il1b*) and *Ccl2* were increased only in VAT of *Lgals3*^−/−^ versus WT mice (Fig. 3a). Immunohistochemical analysis revealed a higher number of F4/80 positive cells in VAT of *Lgals3*^−/−^ animals (Fig. 3b). However, no crown-like structures or dead/dying adipocytes, i.e., negative for the lipid droplet coating protein perilipin-1^21^, were observed (Supplementary Fig. 3). In sera, the concentration of IL-6 was significantly higher in *Lgals3*^−/−^ than in WT mice (Fig. 3c), whereas TNF-α and IL-1β levels were below the limit of detectability in both genotypes. No difference was observed between the two genotypes in the VAT expression of genes coding for the endoplasmic reticulum (ER) stress markers Heat Shock Protein Family A Member 5 (*Hspa5*), C/EBP-Homologous Protein 10 or DNA Damage Inducible Transcript 3 (*Ddit3*), and Tax-Responsive Element-Binding Protein 5 or X-Box Binding Protein 1 (*Xbp1*) (Fig. 3d). The mRNA levels of Collagen Type I Alpha 1 Chain (*Cola1a1*) and Collagen Type I Alpha 1 Chain (*Cola4a1*) were significantly increased only in BAT of *Lgals3*^−/−^ versus WT mice, whereas levels of these and other genes involved in fibrosis (i.e., Fibronectin 1 [*Fn1*] and Collagen Type VI Alpha 1 Chain [*Cola6a1*]) were comparable between the two genotypes in both VAT and SAT (Table 1).

#### Tissue insulin sensitivity

**Figure 4:**
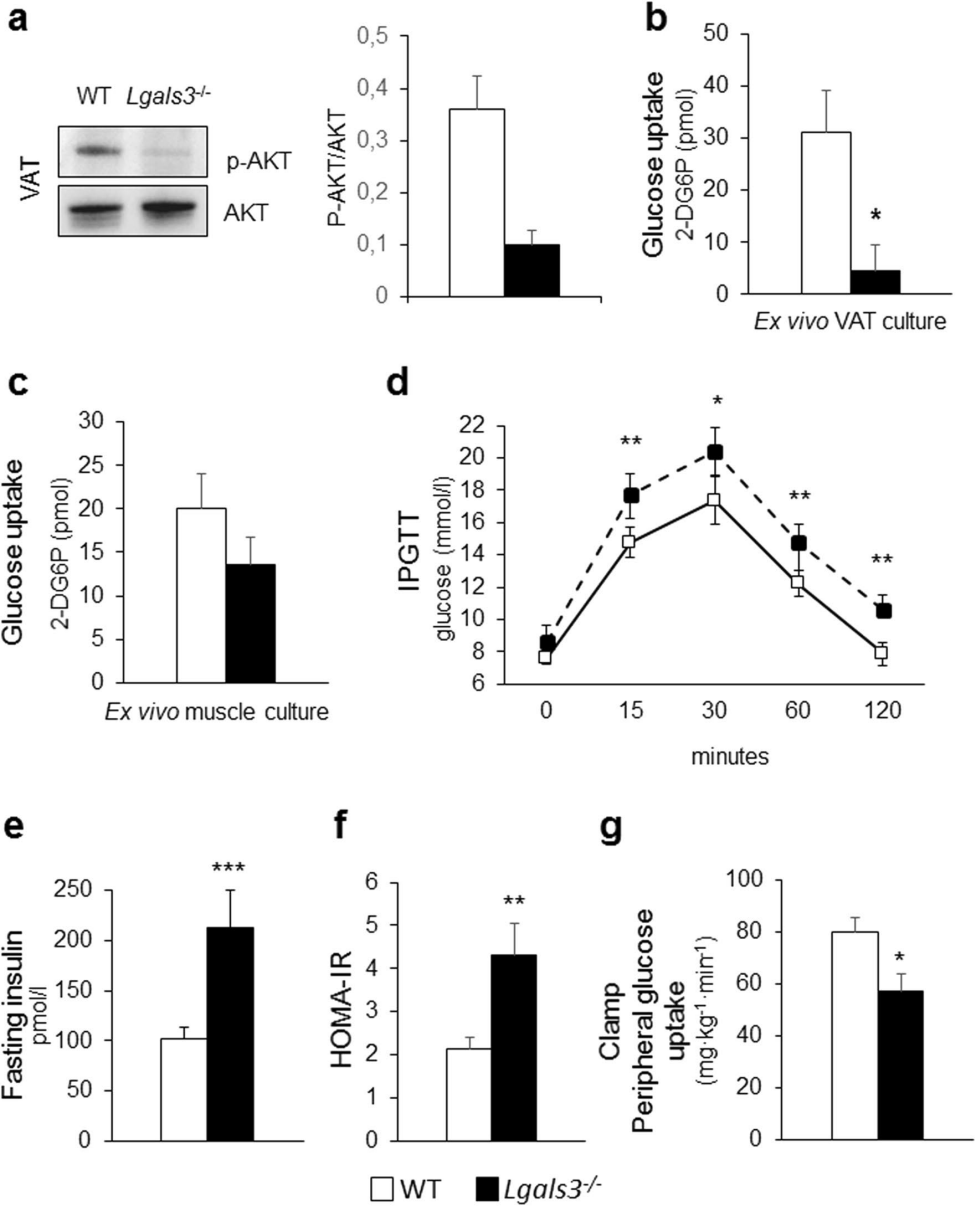
Tissue and systemic insulin sensitivity and glucose homeostasis in 2-month-old WT and *Lgals3*^−/−^ mice. Representative Western blot analysis of phospho-AKT (p-AKT, 60 kDa) and total AKT (AKT, 60 kDa) in protein extracts from WT and *Lgals3*^−/−^ VAT cultures stimulated with insulin and quantification of phospho-AKT (active AKT) relative to total AKT (n = 4 per genotype) (**a**); measurement of glucose uptake in ex vivo cultures of VAT (**b**) and skeletal muscle (**c**) treated with insulin (1 µM) and 2-deoxy-D-glucose (1 mM) from WT or *Lgals3*^−/−^ mice (n = 4 per genotype); IPGTT (n = 5 per genotype) (**d**); fasting insulin levels (**e**), and calculation of HOMA-IR index in WT and *Lgals3*^−/−^ mice (n = 6 per genotype) (**f**); and euglycaemic-hyperinsulinemic clamp in WT and *Lgals3*^−/−^ mice (n = 7 per genotype) (**g**). Values represent the mean ± SD. White bars and squares, continuous line = WT mice; black bars and triangles, dashed line = *Lgals3*^−/−^ mice; *P < 0.05, **P < 0.01 or ***P < 0.001 versus WT.

*Lgals3*^−/−^ mice showed a significant decrease in the gene expression of Glucose Transporter Type 4 or Solute Carrier Family 2 Member 4 (*Slc2a4*) in SAT, VAT, and BAT and Insulin Receptor Substrate 1 (*Irs1*) in SAT, as compared to WT animals (Table 1). Insulin-stimulated AKT phosphorylation and glucose uptake were markedly decreased in VAT from *Lgals3*^−/−^ versus WT mice (Fig. 4a-b), whereas no significant difference in glucose uptake was observed in skeletal muscle (Fig. 4c).

#### Systemic insulin sensitivity and glucose homeostasis

*Lgals3*^−/−^ mice showed unchanged fasting plasma glucose levels with an exaggerated glycemic response to the intraperitoneal glucose tolerance test (IPGTT) (Fig. 4d) as well as increased insulin levels, as compared to WT mice (Fig. 4e). The homeostasis model assessment (HOMA)—insulin resistance (IR) index was also increased in *Lgals3*^−/−^ versus wild type mice (Fig. 4f) and the euglycemic-hyperinsulinemic clamp confirmed the lower whole-body insulin sensitivity in the *Lgals3*^−/−^ animals (Fig. 4g).

### Five-month-old mice

#### AT phenotype

**Figure 5:**
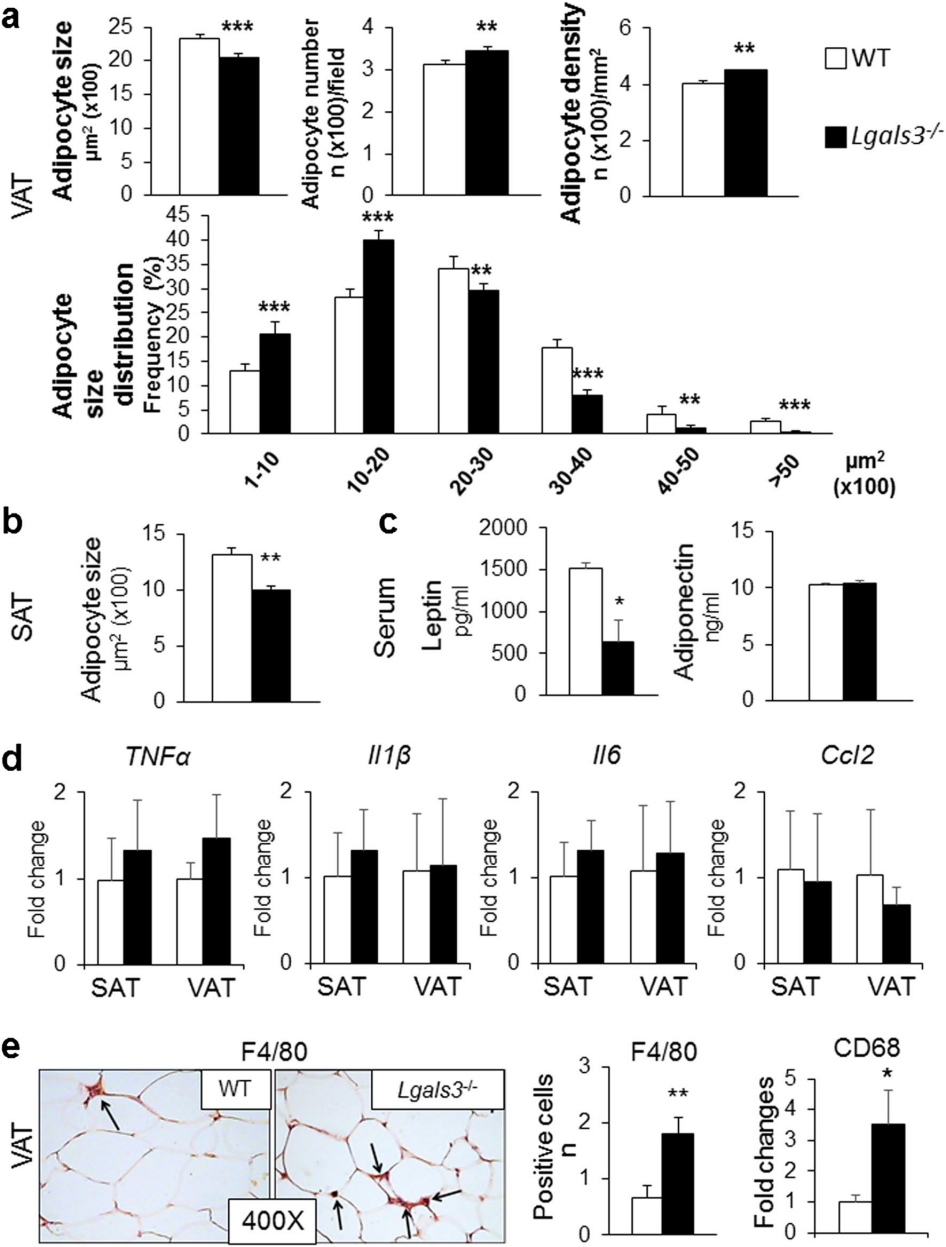
AT phenotype and inflammation in 5-month-old WT and *Lgals3*^−/−^ mice. Quantification of size, number, density and size distribution of adipocytes from VAT (**a**); adipocyte size from SAT (**b**); serum levels of leptin and adiponectin (**c**); qRT-PCR analysis of the inflammatory markers *Tnfa*, *Il1b*, *Il6,* and *Ccl2* in SAT and VAT (**d**); representative immunohistochemical staining for F4/80 (original magnification, 400X; black arrows = macrophages), measurement of F4/80 positive cell number, and RT-PCR analysis of the macrophage marker Cd68 in VAT (**e**) in WT and *Lgals3*^−/−^ mice. Values represent the mean ± SD (n = 6 per genotype). White bars = WT mice; black bars = *Lgals3*^−/−^ mice; *P < 0.05, **P < 0.01 or ***P < 0.001 versus WT.

A substantial improvement in SAT and VAT maturation was observed in *Lgals3*^−/−^ mice, which showed similar gene expression levels of almost all markers of adipogenesis and lipid metabolism to those of WT mice (Table 2). However, adipocyte size remained significantly lower in both VAT (Fig. 5a) and SAT (Fig. 5b), and the number and density of VAT adipocytes as well as the frequency of smaller cells remained significantly higher (Fig. 5a), compared to WT mice. In addition, serum concentration of leptin remained lower in *Lgals3*^−/−^ than in WT mice, and adiponectin levels continued to be similar between the two genotypes (Fig. 5c).

**Table 2:** Adipogenesis, lipid metabolism and fibrosis in adipose tissue of 5-month-old mice.

	SAT		VAT		BAT	
	WT	*Lgals3*^−/−^	WT	*Lgals3*^−/−^	WT	*Lgals3*^−/−^
**Adipogenesis**						
*Cebpb*	1.31 ± 1.63	0.80 ± 0.58	1.34 ± 1.02	0.78 ± 0.47	0.84 ± 0.22	0.99 ± 0.78
*Pparg*	0.67 ± 0.50	0.60 ± 0.21	0.75 ± 0.19	0.61 ± 0.20	1.01 ± 0.02	1.16 ± 0.01*
*Cebpa*	0.80 ± 0.60	0.74 ± 0.31	1.07 ± 0.64	0.93 ± 0.41	1.03 ± 0.04	0.89 ± 0.04*
**Lipolysis**						
*Pnpla2*	1.12 ± 1.19	0.86 ± 0.58	0.40 ± 0.60	0.31 ± 0.45	1.01 ± 0.01	0.99 ± 0.02
**Lipogenesis**						
*Srebf1*	0.80 ± 0.55	0.69 ± 0.37	0.87 ± 0.31	0.84 ± 0.23	0.92 ± 0.11	0.70 ± 0.14
*Acaca*	1.55 ± 2.04	1.39 ± 0.73	0.98 ± 0.53	0.99 ± 0.44	0.79 ± 0.29	0.96 ± 0.44
*Fasn*	0.65 ± 0.51	0.75 ± 0.38	0.97 ± 0.67	0.84 ± 0.43	0.76 ± 0.34	0.61 ± 0.12
*Fabp4*	1.11 ± 0.86	1.05 ± 0.40	4.18 ± 3.68	3.16 ± 3.03	0.99 ± 0.01	0.72 ± 0.16
**Adipokines**						
*Lep*	1.54 ± 2.38	0.58 ± 0.49	1.23 ± 0.76	1.17 ± 0.80	0.67 ± 0.47	1.25 ± 0.78
*Adipoq*	0.84 ± 0.77	0.66 ± 0.31	0.72 ± 0.19	0.75 ± 0.16	1.02 ± 0.02	1.10 ± 0.08
*Cfd*	0.79 ± 0.79	0.72 ± 0.39	0.87 ± 0.32	0.83 ± 0.31	1.00 ± 0.005	0.91 ± 0.11
**Browning**						
*Cidea*	2.91 ± 2.32	1.68 ± 0.75	1.06 ± 1.38	0.13 ± 0.12*	0.92 ± 0.12	0.94 ± 0.07
*Ppara*	1.26 ± 0.69	0.96 ± 0.35	0.88 ± 0.63	0.46 ± 0.28	0.98 ± 0.03	0.98 ± 0.21
*Ucp1*	N/A	N/A	N/A	N/A	1.13 ± 0.18	0.70 ± 0.13*
**Insulin signaling**						
*Slc2a4*	0.89 ± 0.94	0.62 ± 0.35	0.89 ± 0.57	0.64 ± 0.28	0.82 ± 0.25	0.72 ± 0.06
*Insr*	0.62 ± 0.43	0.56 ± 0.35	0.76 ± 0.37	0.84 ± 0.36	1.09 ± 0.13	0.90 ± 0.09
*Irs1*	1.00 ± 0.93	0.87 ± 0.51	1.10 ± 0.34	1.07 ± 0.39	1.54 ± 0.76	1.98 ± 0.73
**Fibrosis**						
*Fn1*	0.45 ± 0.38	0.71 ± 0.33	1.40 ± 0.79	1.86 ± 0.82	1.03 ± 0.04	1.40 ± 0.32
*Cola1a1*	0.26 ± 0.38	0.19 ± 0.10	1.31 ± 0.78	1.68 ± 0.94	1.11 ± 0.16	1.98 ± 0.11†
*Cola4a1*	0.62 ± 0.52	0.54 ± 0.25	1.00 ± 0.32	1.23 ± 0.50	0.99 ± 0.01	1.08 ± 0.36
*Cola6a1*	0.61 ± 2.12	1.46 ± 1.15	1.80 ± 1.71	2.41 ± 2.04	1.18 ± 0.26	1.50 ± 0.49

Expression of genes involved in adipogenesis, white and brown/beige lipid metabolism, insulin signaling, and fibrosis in SAT, VAT and BAT from 5-month-old WT and *Lgals3*^−/−^ mice.

Values represent the mean ± SD (n = 7 per genotype). *P* values calculated by t-test between WT and *Lgals3*^−/−^: *P < 0.05; †*P* < 0.01 and ‡*P* < 0.001 versus the corresponding WT mice.

#### AT and systemic inflammation

Both AT and systemic inflammation reversed almost completely in *Lgals3*^−/−^, with no difference in transcript levels of inflammatory cytokines versus WT animals (Fig. 5d) and serum TNF-α, IL-1β, and IL-6 below the limit of detectability in both genotypes. However, the number of macrophages remained significantly higher in VAT of 5-month-old *Lgals3*^−/−^ versus wild type mice, as assessed by both F4/80-positive cell count and expression of the gene coding for Cluster of Differentiation 68 (*Cd68*) macrophage marker (Fig. 5e).

#### Tissue insulin sensitivity

**Figure 6:**
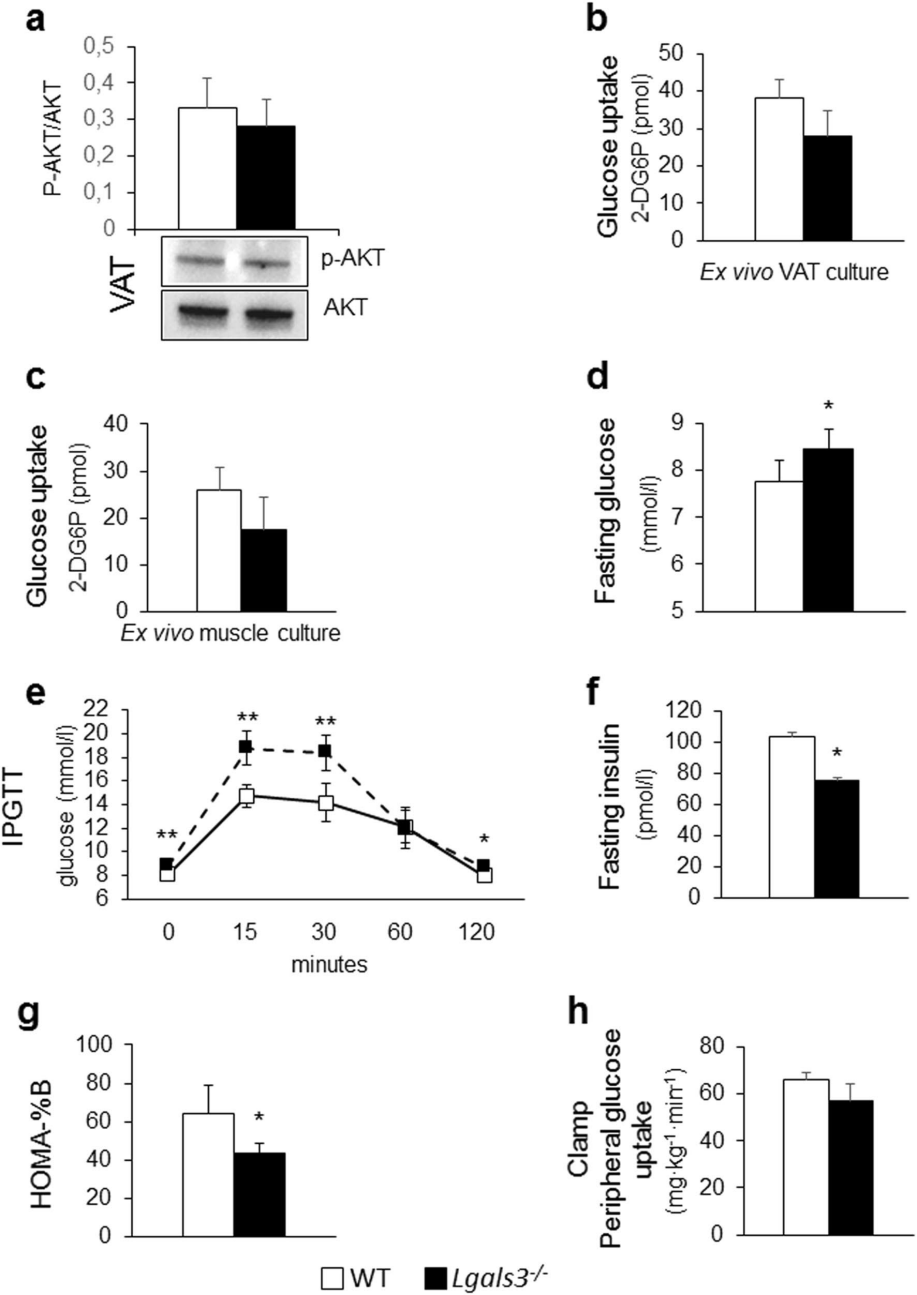
Tissue and systemic insulin sensitivity and glucose homeostasis in 5-month-old WT and *Lgals3*^−/−^ mice. Representative Western blot analysis of phospho-AKT (p-AKT, 60 kDa) and total AKT (AKT, 60 kDa) in protein extracts from VAT cultures stimulated with insulin and quantification of phospho-AKT (active AKT) relative to total AKT (n = 4 per genotype) (**a**); measurement of glucose uptake in ex vivo cultures of VAT (**b**) and skeletal muscle (**c**) treated with insulin (1 µM) and 2-deoxy-D-glucose (1 mM) (n = 4 per genotype); fasting glucose levels (n = 4 per genotype) (**d**); IPGTT (n = 5 per genotype) (**e**); fasting insulin levels (**f**); HOMA-%β (**g**) (n = 6 per genotype); and euglycemic-hyperinsulinemic clamp (n = 7 per genotype) (**h**) in WT and *Lgals3*^−/−^ mice. Values represent the mean ± SD. White bars and squares, continuous line = WT mice; black bars and triangles, dashed line = *Lgals3*^−/−^ mice; *P < 0.05, **P < 0.01 or ***P < 0.001 versus WT.

Insulin-dependent AKT activation and glucose uptake in VAT (Fig. 6a,b) and glucose uptake in muscle (Fig. 6c) were similar in *Lgals3*^−/−^ and WT mice.

#### Systemic insulin sensitivity and glucose homeostasis

Fasting plasma glucose levels were significantly higher (Fig. 6d) and the glucose response to the IPGTT remained abnormal (Fig. 6e) in *Lgals3*^−/−^. Insulin levels decreased markedly in *Lgals3*^−/−^ mice and became significantly lower than in WT animals (Fig. 6f), with a significant reduction in the HOMA-β-cell function (HOMA-%β) index (Fig. 6g). The euglycemic-hyperinsulinemic clamp demonstrated comparable systemic insulin sensitivity in *Lgals3*^−/−^ and WT mice (Fig. 6h).

## Discussion

This study indicates that, under normal feeding conditions, *Lgals3* deletion results in impaired terminal adipogenic differentiation associated with AT dysfunction and inflammation, IR, and altered glucose regulation, in the absence of obvious signs of adipocyte degeneration/death and changes in both the amount and distribution of body fat. From a pathomechanistic point of view, our data support the concept that AT dysfunction limiting tissue plasticity and fat storage capacity may derive not only by a reduced hyperplastic AT response due to defective recruitment and expansion of adipocyte precursors (first step of adipogenesis), but also by a failure of adipocytes to accumulate lipids, due to defective terminal differentiation of pre-adipocytes into fully mature cells (second step of adipogenesis).

Deletion of *Lgals3* led to severely impaired adipogenesis in vitro*.* The SVF cells isolated from *Lgals3*^−/−^ mice failed to undergo growth arrest and terminal differentiation into mature adipocytes following adipogenic induction, with consequent uninterrupted clonal expansion and impaired expression of adipocyte genes and reduced accumulation of triglycerides. This extreme in vitro phenotype translated into a less severe in vivo phenotype, characterized by delayed AT maturation and function, as shown by the reduced expression of genes involved in adipogenesis, browning, lipid metabolism, and endocrine function only in younger (2-month-old) *Lgals3*^−/−^ mice. AT immaturity resulted in decreased adipocyte size and increased percentage of smaller adipocytes, pointing to a reduced fat storing capacity of each individual cell. However, these changes were associated with increased adipocyte number and density depending on a shift in the balance between proliferation/clonal expansion and terminal differentiation in favor of the former, occurring in the absence of galectin-3 during AT development. Therefore, total fat (VAT and SAT) mass was unchanged, suggesting that the overall AT capacity of accumulating lipids was unaffected, at least in the absence of an increased demand for fat storage. Consistently, AT immaturity was not associated with hypertrophy-induced adipocyte damage, increased NEFA release, and, as previously shown in the liver from *Lgals3*^−/−^ mice fed a standard diet, fat diversion to ectopic sites^22^.

Nevertheless, incomplete AT maturation (terminal differentiation) was associated with tissue and systemic inflammation and IR, ultimately leading to impaired glucose homeostasis. Inflammation was detected both in pre-adipocytes derived from *Lgals3*^−/−^ SVF cells and *Lgals3*^−/−^ mice at the AT and systemic level, consistent with previous findings that mesenchymal stem cells and human pre-adipocytes are the primary source of pro-inflammatory cytokines/chemokines^23–25^, particularly IL-6, the levels of which dramatically decrease during adipogenic differentiation^23,24^. Immature adipocytes from the AT may also have contributed to the increased plasma IL-6 levels of 2-month-old *Lgals3*^−/−^ mice, as previously demonstrated in leptin-deficient obese (*ob/ob*) mice^24^. In parallel with the defective maturation and increased expression of IL-6, ex vivo glucose uptake and insulin sensitivity were reduced in AT, whereas they were unaffected in muscle, consistent with previous observations in *Lgals3*^−/−^ mice^26^. These changes at the AT level resulted in an impairment of whole-body insulin sensitivity and glucose homeostasis, which were associated with increased circulating levels of IL-6, in keeping with the findings that serum IL-6 levels are elevated in IR and metabolic disorders^27,28^ and that chronic exposure to IL-6 produces muscle IR^29^. Though whole-body IR as measured by the euglycemic-hyperinsulinemic clamp technique is mainly accounted for by a reduction in muscle insulin sensitivity, the unchanged NEFA levels despite twice-higher insulin concentrations provided indirect evidence that the AT of *Lgals3*^−/−^ mice was insulin resistant also in vivo, consistent with the ex vivo findings.

In vivo, AT maturation was only delayed, since at 5 months of age the gene expression profile was not different between *Lgals3*^−/−^ and coeval wild type mice. In parallel, AT and systemic inflammation and IR were no longer observed in 5-month-old *Lgals3*^−/−^ mice, further supporting the relationship of these changes with the impaired AT maturation observed in younger animals. Conversely, the number of macrophages continued to be elevated in the VAT of *Lgals3*^−/−^ mice, a finding possibly related to the lack of the specific regulatory functions of *Lgals3* in macrophage homeostasis^30,31^. In addition, the comparable increase in AT macrophages from *Lgals3*^−/−^ versus wild type animals at 2 and 5 months of age further supports the concept that immature adipocytes are the main source of the increased cytokine levels observed in 2-month-old *Lgals3*^−/−^ mice. Despite maturation of adipocytes, resolution of inflammation, and restoration of insulin sensitivity, older *Lgals3*^−/−^ mice showed persistently abnormal glucose metabolism associated with reduced insulin levels and HOMA-%B, suggesting that *Lgals3* ablation might impair also β-cell functional reserve during aging.

Overall our data indicate that the concept of adiposopathy should be extended to normal feeding conditions, under which an AT dysfunction due to a defect of terminal adipogenesis may directly cause inflammation and IR at the tissue and systemic level, without inducing adipocyte degeneration/death and fat diversion to ectopic sites, consistent with the unchanged total fat mass. Conversely, in response to a high-fat intake, such an adipogenic defect may lower the threshold of critical volume beyond which adipocyte injury, fat diversion, and the detrimental downstream events occur^9^. This concept is consistent with the increased susceptibility of *Lgals3*^−/−^ mice to IR, inflammation, and diabetes induced by a high-fat diet^18,19^.

The results of our study have important clinical implications, as they suggest that a genetic background that is associated with an impaired ability of pre-adipocytes to terminal differentiate into functional mature cells without affecting pre-adipocyte recruitment may underlie an increased susceptibility to develop abnormalities of insulin sensitivity and glucose regulation. Consistently, a growing body of evidence from human and experimental obesity indicates that adiposopathy is mainly characterized by a defect in the hypertrophic response of newly formed adipocytes rather than by an impaired recruitment and proliferation of adipocyte precursors^32–36^.

Our results may reconcile the apparently discordant findings of defective adipogenesis, unchanged body weight, and impaired glucose regulation previously reported in *Lgals3*^−/−^ mice fed a standard diet^17–19^. Nevertheless, there are some differences with previous studies using this mouse model, which however were also in contrast between each other for several findings^17–20^.

The main strength of our work is the use of the *Lgals3*^−/−^ mouse model, which is characterized by both impaired adipocyte differentiation and altered glucose regulation and is therefore suited to test the study hypothesis. A weakness is the use of total body instead of AT-specific *Lgals3*^−/−^ mice, which did not allow to rule out a background effect of whole-body deletion of *Lgals3* especially on inflammation, which is modulated by this lectin^13,14^, but also on IR and glucose homeostasis. Indeed, as discussed above, the persistence of altered glucose regulation despite AT maturation in 5-month-old *Lgals3*^−/−^ mice might suggest that *Lgals3* ablation directly affects the β-cell. However, the strict temporal relationship between AT maturation and function and systemic IR and inflammation, with all these changes being observed at 2 months and no longer detected at 5 months of age, strongly support a major role for *Lgals3* deficiency at the AT level in the pathogenesis of metabolic derangement. Other limitations include the lack of mechanistic studies explaining the delayed AT maturation and the pancreatic defect observed in *Lgals3*^−/−^ mice, which require further studies. However, unrevealing the mechanisms underlying the effect of *Lgals3* ablation on AT and other metabolic organs was beyond the scope of our work.

In conclusion, our data indicate that *Lgas3* deletion results in impaired terminal differentiation (maturation) of pre-adipocytes, which might directly affect insulin sensitivity and increase susceptibility to type 2 diabetes in the absence of an increased demand for fat storage and, hence, independently of increased fat accumulation and redistribution. This implies that defective adipogenesis is a central mechanism underlying IR and susceptibility to diabetes, thus providing the conceptual framework for designing and testing new molecules targeting AT adipogenic capacity.

## Methods

### Design

The study protocol was approved by the National Ethics Committee for Animal Experimentation of the Italian Minister of Health (D.lgs. 26/2014, Act n. 184) and included in vitro, in vivo, and ex vivo studies using 5-week-old female *Lgals3*^−/−^ and the corresponding C57BL/6J WT mice (gift of Daniel K Hsu & Fu-Tong Liu, Department of Dermatology, University of California-Davis, Sacramento, CA, USA).

#### In vitro studies

The SVF cells were isolated from SAT of WT and *Lgals3*^−/−^ mice. Briefly, tissue was washed with PBS, mechanically disrupted, and enzymatically digested in a buffer containing 1.5 mg/ml collagenase type II, 0.5% BSA, and 15 mM HEPES (Merck KGaA, Darmstadt, Germany) for 45 min at 37 °C and 150 rpm. The disassociated tissue was filtered and centrifuged at 700 × *g* for 10 min to pellet SVF cells, which were then plated in DMEM/F12 supplemented with 10% FBS and 1% penicillin/streptomycin (Thermo Fisher Scientific, Waltham, MA, USA).

For *Lgals3* knockdown experiments in mature adipocytes, murine 3T3-L1 pre-adipocytes (ATCC, Manassas, VA, USA) were induced to differentiate into fully mature adipocytes as per vendor’s protocol. At day 7 post-induction, adipocytes were reverse transfected using Silencer Select siRNA to *Lgals3* and irrelevant scrambled siRNA as control (Thermo Fisher Scientific) at day 7 post-induction using DharmaFECT Duo Transfection Reagent (Healthcare Dharmacon Inc., Lafayette, CO, USA).

#### In vivo studies

The animals were housed in single cages in a germ-free stabularium and cared according to the “Animal Research: Reporting of In Vivo Experiments” (ARRIVE) guidelines (https://www.nc3rs.org.uk/arrive-guidelines) and national laws and regulations. Mice received water and food ad libitum. Body weights and fasting plasma glucose levels were measured monthly, together with food intake.

Part of the mice were subjected to an IPGTT (n = 6 per age and genotype) or a euglycemic-hyperinsulinemic clamp (n = 7 per age and genotype). The remaining mice (n = 6 per age and genotype) were starved for 4 h, then blood samples were collected, the animals were euthanized by cervical dislocation, and inguinal SAT, gonadal VAT, and interscapular BAT were sampled, weighed, and fixed in phosphate buffered 4% formaldehyde solution and processed for morphometric and immunohistochemical analysis or frozen in liquid nitrogen and stored at − 80 °C until analysis of gene expression by qRT-PCR.

#### Ex vivo studies

Samples of VAT and gastrocnemius muscle were obtained from 4 mice per age and genotype, washed in PBS, and cultured for 2 h in RPMI containing penicillin and streptomycin (Thermo Fisher Scientific). Then, tissues were stimulated with 1 µM insulin (Merck KGaA) or vehicle for 20 min and processed for the assessment of glucose uptake or, in case of VAT, frozen and stored at − 80 °C for Western blot analysis.

### Measurements

#### Adipogenic differentiation

The SVF cells were cultured for 14 days in complete DMEM/F12 medium (Thermo Fisher Scientific) supplemented with 50 μM indomethacine, 0.5 μM hydrocortisone, 0.5 mM isobutylmethylxanthine, and 5 μg/ml insulin (Merck KGaA).

Lipid accumulation was detected using Oil-Red-O staining (O0625, Sigma-Aldrich, St.Louis, MO, USA) with the aid of the image analysis system Optimas 6.5 (Bioscan, Washington DC, USA).

Cell proliferation was assessed by measuring BrdU incorporation using an Eloisa kit from Cell Biolabs (San Diego, CA, USA) following the manufacturer’s instructions.

#### Biochemistry and ELISA

Fasting blood samples were analyzed for glucose by an automated colorimetric instrument (Glucocard SM, Menarini, Florence, Italy) and insulin by ELISA (Ultrasensitive Mouse Insulin ELISA kit, Mercodia AB, Uppsala, Sweden); the HOMA-IR and HOMA-%β indexes were then calculated from glucose and insulin.

Levels of NEFAs were measured with a colorimetric Kit (MAK044, Merck KGaA). Serum levels of TNF-α, IL-1β, IL-6, leptin, and adiponectin were assessed using ELISA kits from R&D Systems (Minneapolis, MN, USA) and ENZO Life Sciences (Farmingdale, NY, USA) for leptin.

#### Histology, morphometry and Immunohistochemistry

Sections of VAT and SAT were stained with hematoxylin–eosin for histological examination. Adipocyte size in VAT and SAT was evaluated by computer assisted image analysis (Optimas 6.5) on five serial sections viewed at 100X using a DIALUX 20EB Microscope (Leitz, Wetzlar, Germany) and a Pro-Series High performance CCD Camera (Immagini e Computer, Milan, Italy). Ten random fields of 0.78 mm^2^ per section, automatically selected by means of a step motor, were analyzed. Data were first averaged per section and then per animal.

The number of infiltrating macrophages was measured in five AT sections immunolabelled for the murine macrophage marker F4/80; twenty random, automatically selected 0.125 mm^2^ fields per section were analyzed. Perilipin-1 immunostaining was performed to identify dead/dying adipocytes in areas of accumulation of F4/80 positive cells. The antibodies used for F4/80 and perilipin-1 immunohistochemistry are reported in Supplementary Table 1.

#### RNA isolation and qRT-PCR

Total RNA was extracted from SAT and VAT using an RNA purification kit (QIAGEN, Hilden, Germany) and from BAT and cell cultures using TRIzol Reagent (Thermo Fisher Scientific).

qRT-PCR was carried out in 20 µl reactions using a StepOne RT-PCR instrument (Applied Biosystems, Monza, Italy)^37^. The expression of the following genes was assessed using the TaqMan Gene Expression Assays (Applied Biosystems) reported in Supplementary Table 2: *Lgals3*; the pre-adipocyte marker and adipogenesis inhibitor *Dlk1*; the adipogenic transcription factors *Cebpa*, *Cebpb* and *Pparg*; the lipolytic enzyme *Pnpla2*; the lipogenic factors *Srebf1*, *Acaca*, *Fasn*, and *Fabp4*; the adipokines *Lep*, *Adipoq*, and *Cfd*; the markers of browning *Cidea*, *Ppara* and, in BAT, *Ucp1*; the cell cycle regulator *Ccnd1*; the insulin signaling mediators Insulin Receptor (*Irs1*), *Irs1*, and *Slc2a4*; the inflammatory cytokines *Tnfa*, *Il1b*, *Il6*, and *Ccl2*; the macrophage marker *Cd68*; the ER stress markers *Hspa5*, *Ddit3*, and *Xbp1*; and the fibrosis markers *Fn1*, *Cola1a1*, *Cola4a1*, and *Cola6a1*. Amplifications were normalized to the β-actin gene (*Actb*) and quantitation was performed using the ΔΔCT calculation, where results were expressed as arbitrary units or fold of control mean (WT mouse and untreated WT cells) to control for unwanted sources of variation.

#### Euglycemic-hyperinsulinemic clamp and IPGTT

For euglycemic-hyperinsulinemic clamp^38,39^, mice underwent surgery for the positioning of catheters. Three-to-five days later, after a 6-h fast, a primed continuous (18.0 mU·kg^-1^·min^-1^) infusion of human insulin (Actrapid, Novo Nordisk, Copenhagen, Denmark) was started simultaneously with a variable infusion of 20% dextrose to maintain glucose concentration at 80–100 mg dL^-1^. Blood samples were taken at time 0 and at 10-min intervals thereafter for at least 2 h to measure glucose concentration and adjust dextrose infusion rates. Insulin sensitivity (rate of peripheral glucose uptake [mg·kg^-1^·min^-1^]) was calculated from average glucose concentrations and dextrose infusion rates during the last 30 min of the steady-state clamp period.

For IPGTT, after a 6-h fast, blood was drawn to measure glucose levels before and 15, 30, 60, and 120 min after intraperitoneal injection of 1 g glucose per kg body weight (Merck KGaA).

#### Glucose uptake

Glucose uptake was assessed in insulin- or vehicle-stimulated VAT and gastrocnemius samples using the Glucose Uptake Colorimetric Assay Kit (MAK083, Merck KGaA), following the manufacturer’s instructions.

#### Western blot analysis

VAT samples stimulated with insulin or vehicle were homogenized in RIPA buffer supplemented with protease and phosphatase inhibitors.

Total and phosphorylated Akt protein levels were assessed by Western blot analysis^40^ using the antibodies reported in Supplementary Table 1. The protein bands were detected and analyzed by using the ChemiDoc XRS** + **System with Image Lab image acquisition and analysis software included (Bio-Rad laboratories, Hercules, CA, USA). Results were normalized using the Stain-Free technology (Bio-Rad laboratories) and Akt phosphorylation was expressed as phosphorylated:total Akt ratio.

### Statistical analysis

Results were expressed as means ± SD. Statistical significance was evaluated by Student's t test or the corresponding Mann–Whitney U test, for nonparametric variables.

A *P*-value < 0.05 was considered significant. All statistical tests were performed on raw data.

## Supplementary information

Supplementary Information.

## Abbreviations

AT: Adipose tissue
IR: Insulin resistance
PPAR: Peroxisome proliferator-activated receptor
*Lgals3*^−^^/^^−^: Galectin 3 knockout
WT: Wild-type
SVF: Stromal vascular precursor
SAT: Subcutaneous AT
*Dlk1*: Preadipocyte factor 1 or Delta Like Non-Canonical Notch Ligand gene
*Pparg*: PPARγ gene
CEBP: Ccaat-Enhancer-Binding Protein
*Cebpb*: CEBP β gene
*Pnpla2*: Adipose Triglyceride Lipase or Patatin Like Phospholipase Domain Containing 2 gene
*Srebf1*: Sterol Regulatory Element Binding Transcription Factor 1 gene
*Acaca*: Acetyl-CoA Carboxylase α gene
*Fasn*: Fatty Acid Synthase gene
*Adipoq*: Adiponectin gene
IL: Interleukin
*Il6*: IL-6 gene
*Ccl2*: Monocyte chemoattractant protein 1 or C–C Motif Chemokine Ligand 2 gene
*Ccnd1*: Cyclin D1 gene
BrdU: 5-Bromo-2′-deoxyuridine
VAT: Visceral AT
*Cebpa*: CEBP α gene
*Fabp4*: Fatty acid binding protein 4 gene
*Lep*: Leptin gene
*Cdf*: Adipsin or Complement Factor D gene
BAT: Brown AT
*Cidea*: Cell Death-Inducing DFFA-Like Effector A gene
*Ppara*: PPARα gene
*Ucp1*: Uncoupling Protein 1 gene
NEFAs: Non-esterified fatty acids
TNF: Tumor necrosis factor
*Tnfa*: Tumor necrosis factor -α gene
*Il1b*: IL-1β gene
ER: Endoplasmic reticulum
*Hspa5*: Heat Shock Protein Family A Member 5 gene
*Ddit3*: C/EBP-Homologous Protein 10 or DNA Damage Inducible Transcript 3 gene
*Xbp1*: Tax-Responsive Element-Binding Protein 5 or X-Box Binding Protein 1 gene
*Fn1*: Fibronectin 1 gene
*Cola1a1*: Collagen Type I Alpha 1 Chain gene
*Cola4a1*: Collagen Type IV Alpha 1 Chain gene
*Cola6a1*: Collagen Type VI Alpha 1 Chain gene
*Slc2a4*: Glucose Transporter Type 4 or Solute Carrier Family 2 Member 4 gene
*Irs1*: Insulin Receptor Substrate 1 gene
*Insr*: Insulin Receptor gene
IPGTT: Intraperitoneal glucose tolerance test
HOMA: Homeostasis model assessment
HOMA-IR: HOMA-insulin resistance
*Cd68*: Cluster of differentiation 68
HOMA-%β: HOMA-β-cell function
*Actb*: β-Actin gene
