# Supplementary material for: Galectin-3 gene deletion results in defective adipose tissue maturation and impaired insulin sensitivity and glucose homeostasis
Source: Sci Rep. 2020 Nov 18;10:20070. doi: 10.1038/s41598-020-76952-z (PMC7675972; doi:10.1038/s41598-020-76952-z)
Supplement: Supplementary file 1 — Supplementary Information. [file 41598_2020_76952_MOESM1_ESM.pdf]

# Galectin-3 gene deletion results in defective adipose tissue maturation and impaired insulin sensitivity and glucose homeostasis

Claudia Blasetti Fantauzzi, Carla Iacobini, Stefano Menini, Martina Vitale, Gian Pio Sorice, Teresa Mezza, Saverio Cinti, Andrea Giaccari, and Giuseppe Pugliese

**Supplementary Table 1.** Primary and secondary antibody list used in Western blot and immunohistochemistry.

| Primary Antibody | Species | Dilution | Company | Catalog number |
|---|---|---|---|---|
| **Akt 1/2/3** | rabbit monoclonal | 1:1,000 | Abcam (Cambridge, UK) | AB 179463 |
| **Akt p** | rabbit polyclonal | 1:1,000 | Abcam (Cambridge, UK) | AB 66138 |
| **Galectin-3** | mouse monoclonal | 1:5000 | Abcam (Cambridge, UK) | AB 2785 |
| **β-actin** | mouse monoclonal | 1:10,000 | Merck KGaA (Darmstadt, Germany) | A5441 |
| **F4/80** | rat monoclonal | 1:200 | Novus Biologicals (Littleton, CO, USA) | NB 600-404 |
| **Perilipin-1** | goat polyclonal | 1:40 | Abcam (Cambridge, UK) | AB 61682 |
| **Secondary Antibody** | **Species** | **Dilution** | **Company** | **Catalog number** |
| HRP-conjugated anti rabbit Ig | goat | 1:5,000 | Dako (Glostrup, Denmark) | P0448 |
| HRP-conjugated anti mouse Ig | goat | 1:2,500 | Dako (Glostrup, Denmark) | P0447 |
| Anti-rat IgG H&L (Biotin) | goat | 1:400 | Abcam (Cambridge, UK) | AB 6844 |
| Anti-goat IgG H&L (Biotin) | rabbit | 1:500 | Abcam (Cambridge, UK) | AB 6740 |

**Supplementary Table 2.** TaqMan Assays used in quantitative real time PCR.

| Mouse TaqMan Assay | Code |
|---|---|
| *Lgals3* | Mm00802901_m1 |
| *Dlk1* | Mm00494477_m1 |
| *Cebpa* | Mm00514283_s1 |
| *Cebpb* | Mm00843434_s1 |
| *Pparg* | Mm01184322_m1 |
| *Pnpla2* | Mm00503040_m1 |
| *Srebf1* | Mm00550338_m1 |
| *Acaca* | Mm01304257_m1 |
| *Fasn* | Mm00662319_m1 |
| *Fabp4* | Mm00445878_m1 |
| *Lep* | Mm00434759_m1 |
| *Adipoq* | Mm00456425_m1 |
| *Cfd* | Mm01143935_g1 |
| *Cidea* | Mm00432554_m1 |
| *Ppara* | Mm00440939_m1 |
| *Ucp1* | Mm01244861_m1 |
| *Ccnd1* | Mm00432359_m1 |
| *Insr* | Mm01211875_m1 |
| *Irs1* | Mm01278327_m1 |
| *Slc2a4* | Mm01245502_m1 |
| *Tnfa* | Mm00443258_m1 |
| *Il1b* | Mm00434228_m1 |
| *Il6* | Mm00446190_m1 |
| *Ccl2* | Mm00441242_m1 |
| *Cd68* | Mm03047343_m1 |
| *Adgre1* | Mm00802529_m1 |
| *Fn1* | Mm01256744_m1 |
| *Cola1a1* | Mm00801666_g1 |
| *Cola4a1* | Mm01210125_m1 |
| *Cola6a1* | Mm00711678_m1 |
| *Actb* | Mm00607939_s1 |

*Lgals3*=galectin-3; *Dlk1*=Preadipocyte factor 1 or Delta Like Non-Canonical Notch Ligand; *Cebpa*=Ccaat-Enhancer-Binding Protein α; *Cebpb*=Ccaat-Enhancer-Binding Protein β; *Pparγ*=peroxisome proliferator-activated receptor γ; *Pnpla2*=Adipose Triglyceride Lipase or Patatin Like Phospholipase Domain Containing 2; *Srebf1*=Sterol Regulatory Element Binding Transcription Factor 1; *Acaca*=Acetyl-CoA Carboxylase α; *Fasn*=Fatty Acid Synthase; *Fabp4*=Fatty Acid Binding Protein 4; *Lep*=Leptin; *Adipoq*=Adiponectin; *Cfd*=Adipsin or Complement Factor D; *Cidea*=Cell Death-Inducing DFFA-Like Effector A; *Ppara*= peroxisome proliferator-activated receptorα; *Ucp1*=Uncoupling Protein 1; *Ccnd1*=Cyclin D1; *Insr*=Insulin Receptor; *Irs1*=Insulin Receptor Substrate 1; *Slc2a4*=Glucose Transporter Type 4 or Solute Carrier Family 2 Member 4; *Tnfa*=tumor necrosis factor α; *Il1b*=interleukin 1β; *Il6*=interleukin 6; *Ccl2*= Monocyte Chemoattractant Protein-1 or C-C Motif Chemokine Ligand 2 (Ccl2); *Cd68*= Cluster of Differentiation 68; *Adgre1*=F4/80 or Adhesion G Protein-Coupled Receptor E1; *Fn1*=Fibronectin 1; *Cola1a1*=Collagen Type I Alpha 1 Chain; *Cola4a1*=Collagen Type IV Alpha 1 Chain; *Cola6a1*=Collagen Type VI Alpha 1 Chain; *Actb* =β-actin.

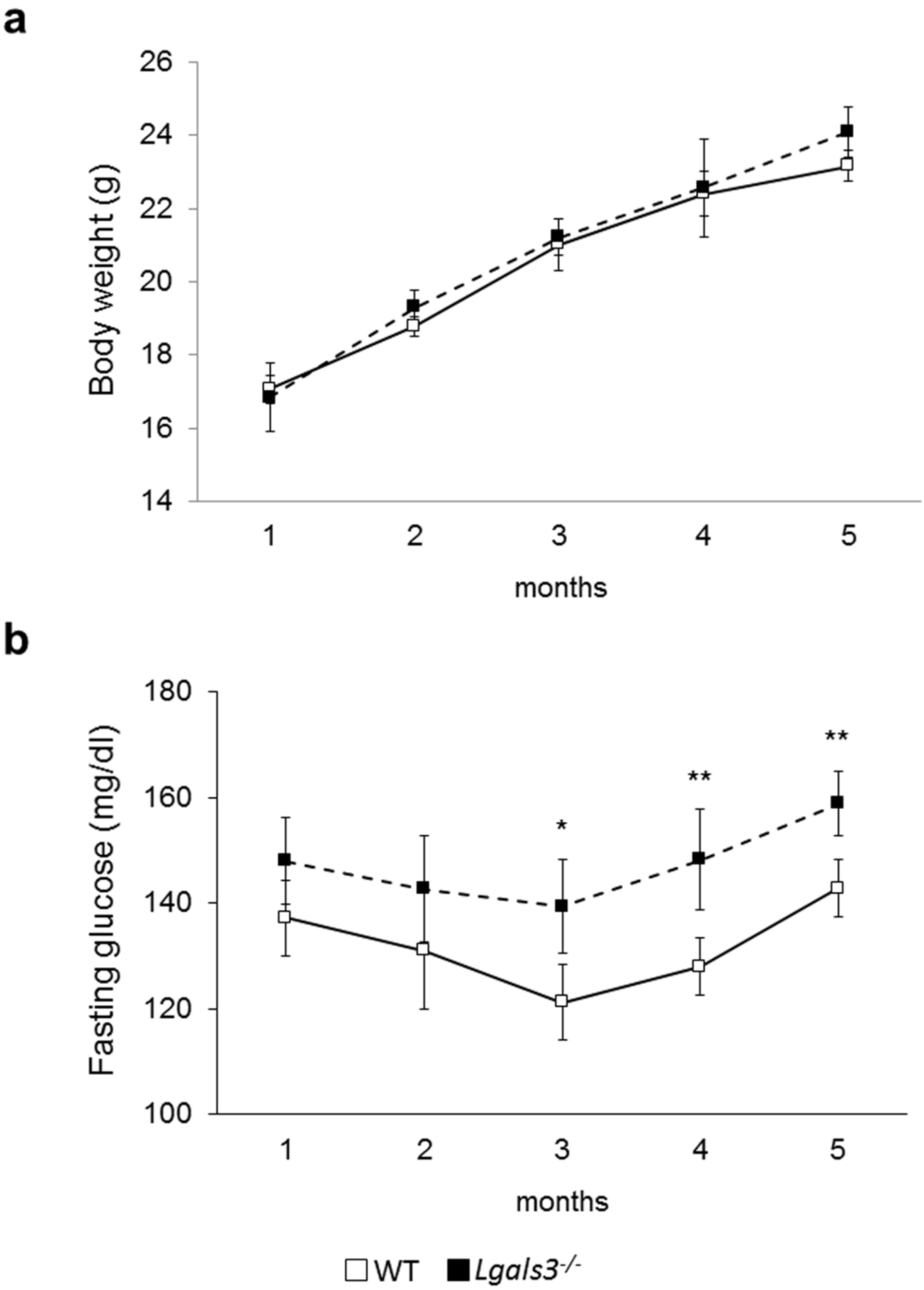


**Supplementary Figure 1. Trends in body weight and fasting plasma glucose in WT and *Lgals3*$^{-/-}$ mice.** Changes in body weight (**a**) and fasting plasma glucose levels (**b**) in WT and *Lgals3*$^{-/-}$ mice throughout the study period. Values represent the mean±SD (n=17 per genotype). *P<0.05 or **P<0.01 versus WT.

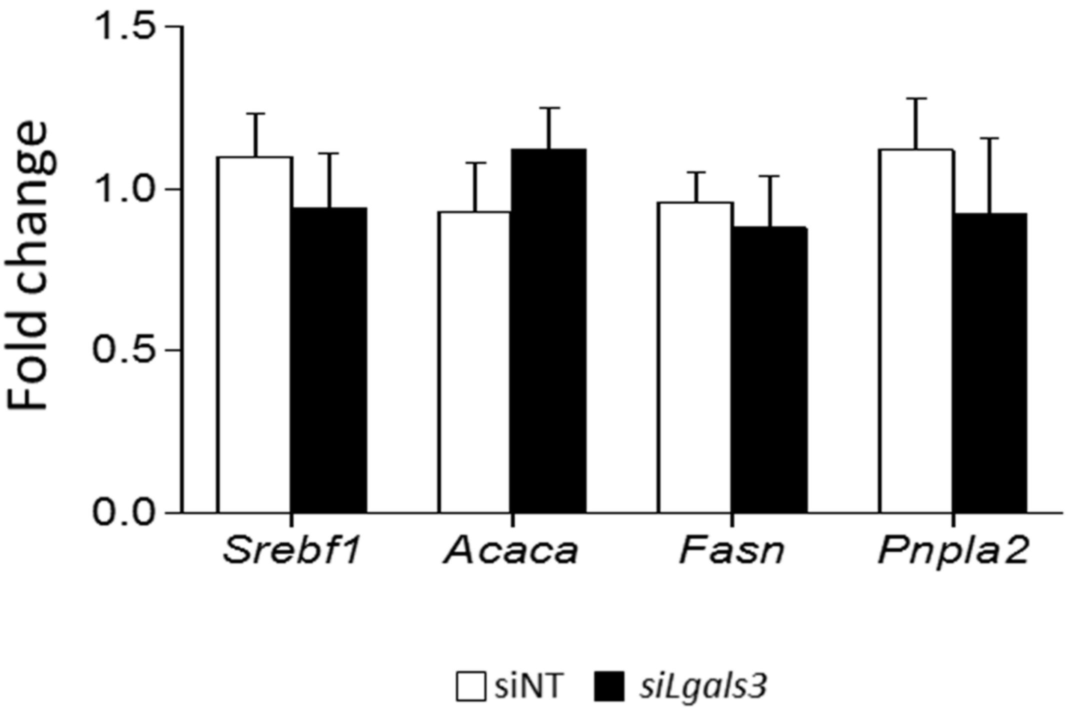


**Supplementary Figure 2. Cellular lipid metabolism in 3T3-L1 silenced for *Lgals3*.** RT-PCR analysis of genes involved in lipid metabolism (*Srebf1*, *Acaca*, *Fasn*, and *Pnpl+a2*) from 3T3-L1 siNT control and si*Lgals3* cells. *P<0.05, **P<0.01 or ***P<0.001 vs WT.  siNT = non-target control.

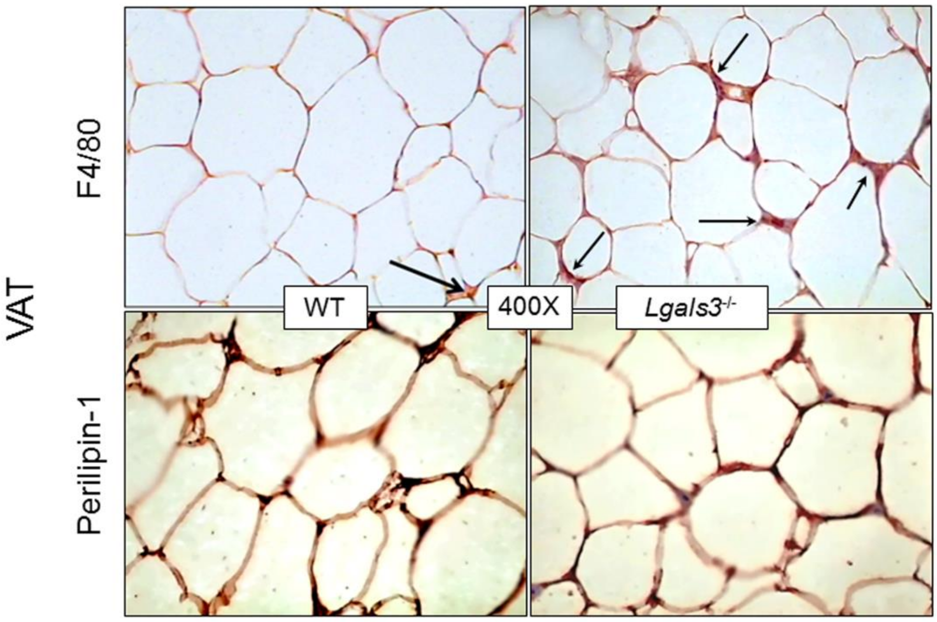


**Supplementary Figure 3. F4/80 and perilipin-1 staining in 2-month-old wild type and *Lgals3*$^{-/-}$ mice.** Serial sections of VAT from representative 2-month-old WT and *Lgals3*$^{-/-}$ mice immunolabelled for F4/80, a murine macrophage marker, and perilipin-1, a lipid droplet coating protein, using the antibodies reported in Supplementary Table 1. There are no perilipin negative (dead) adipocytes in WT and *Lgals3*$^{-/-}$ mice.  Black arrows = macrophages.

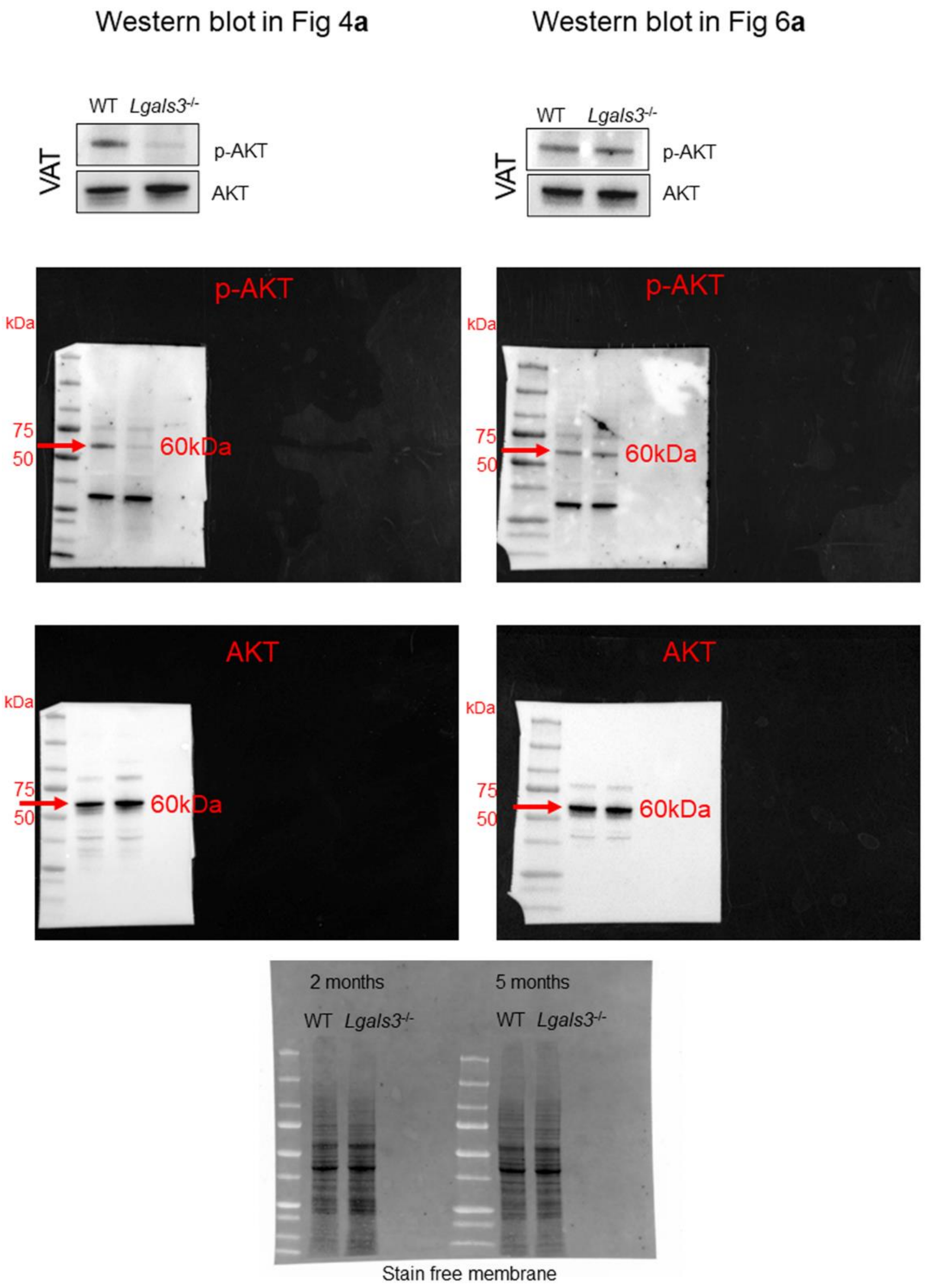


**Supplementary Figure 4.** Unedited gel images of Western blot analysis of phospho-AKT (p-AKT, 60kDa) and total AKT (AKT, 60 kDa) in protein extracts from WT and *Lgals3*$^{-/-}$ VAT cultures stimulated with insulin. Normalization for total protein content was performed on the same Stain-Free gel (lower box) using Stain-Free technology (Bio-Rad laboratories).
